# Discriminating Micropathogen Lineages and Their Reticulate Evolution through Graph Theory-Based Network Analysis: The Case of *Trypanosoma cruzi*, the Agent of Chagas Disease

**DOI:** 10.1371/journal.pone.0103213

**Published:** 2014-08-22

**Authors:** Sophie Arnaud-Haond, Yann Moalic, Christian Barnabé, Francisco José Ayala, Michel Tibayrenc

**Affiliations:** 1 IFREMER (Institut Français de Recherche pour l'Exploitation de la Mer) - Département Ecosystèmes Marins Exploités, Sète, France; 2 Interactions hôte-vecteur-parasite dans les maladies dues aux Trypanosomatidés, INTERTRYP (IRD-CIRAD), Montpellier, France; 3 Department of Ecology and Evolutionary Biology, University of California Irvine, Irvine, California, United States of America; 4 Maladies Infectieuses et Vecteurs Ecologie, Génétique, Evolution et Contrôle, MIVEGEC (IRD 224-CNRS 5290-UM1-UM2), Montpellier, France; Albert Einstein College of Medicine, United States of America

## Abstract

Micropathogens (viruses, bacteria, fungi, parasitic protozoa) share a common trait, which is partial clonality, with wide variance in the respective influence of clonality and sexual recombination on the dynamics and evolution of taxa. The discrimination of distinct lineages and the reconstruction of their phylogenetic history are key information to infer their biomedical properties. However, the phylogenetic picture is often clouded by occasional events of recombination across divergent lineages, limiting the relevance of classical phylogenetic analysis and dichotomic trees. We have applied a network analysis based on graph theory to illustrate the relationships among genotypes of *Trypanosoma cruzi*, the parasitic protozoan responsible for Chagas disease, to identify major lineages and to unravel their past history of divergence and possible recombination events. At the scale of *T. cruzi* subspecific diversity, graph theory-based networks applied to 22 isoenzyme loci (262 distinct Multi-Locus-Enzyme-Electrophoresis -MLEE) and 19 microsatellite loci (66 Multi-Locus-Genotypes -MLG) fully confirms the high clustering of genotypes into major lineages or “near-clades”. The release of the dichotomic constraint associated with phylogenetic reconstruction usually applied to Multilocus data allows identifying putative hybrids and their parental lineages. Reticulate topology suggests a slightly different history for some of the main “near-clades”, and a possibly more complex origin for the putative hybrids than hitherto proposed. Finally the sub-network of the near-clade *T. cruzi* I (28 MLG) shows a clustering subdivision into three differentiated lesser near-clades (“Russian doll pattern”), which confirms the hypothesis recently proposed by other investigators. The present study broadens and clarifies the hypotheses previously obtained from classical markers on the same sets of data, which demonstrates the added value of this approach. This underlines the potential of graph theory-based network analysis for describing the nature and relationships of major pathogens, thereby opening stimulating prospects to unravel the organization, dynamics and history of major micropathogen lineages.

## Introduction

At odds with theoretical predictions about their considerable cost, sexuality and recombination are ubiquitous, at least in metazoa. This is the “paradox of sex”. Theoretical models may overlook important factors such as drift, and spatio-temporal variation of selective pressure, that could reduce the advantages conferred by recombination [1]–[3]. Recombination may be positively selected by breaking down the negative associations generated by random drift [4], and may allow the emergence of unique multilocus genotypes gathering advantageous alleles that arose in different individuals at the same locus [5] or at other loci [6]. It may be noted, however, that several authors do not consider that generation of new, better-adapted multilocus associations is the main reason for maintenance of genetic recombination in micropathogens, be they viruses [7] or bacteria [8]. They have rather proposed that recombination is a side effect of other evolutionary mechanisms, such as DNA repair for example [9].

A large number of microbial pathogens owe their persistence in a changing environment to new epistatic combinations optimized for pathogenic properties or drug resistance arising from either regular or sporadic recombination events [10], [11]. Microbial pathogens offer a large spectrum of clonal *versus* recombinant reproductive modes, from a high rate of systematic outcrossing by normal meiosis (or “symmetric recombination”) as it is the case in some African populations of the malaria agent (*Plasmodium falciparum* [12]), to predominantly clonal evolution for the agent of Chagas disease (*Trypanosoma cruzi*, [13]). Clonality here is understood as encompassing all situations where genetic recombination is severely restrained and is not frequent enough to break the prevalent pattern of clonal evolution [14]. Yet, even in predominantly clonal species, some sort of recombination may be expected even at very low frequency [2]. The understanding of the evolutionary dynamics of pathogenicity and drug resistance requires knowledge of the distribution of the genes implicated, and of the mechanisms that drive their dynamics. The pattern and rate of recombination should also be known in order to map the genome and the allelic variants involved in pathenogenicity variation and drug resistance [15], as well as for the epidemiologic characterization of strains and lineages by Multilocus Genotyping (MLG), Multilocus Sequence Typing (MLST) or other markers [16]–[18]. Wherever clonality predominates, genotypic associations are expected to be stable in space and time, whereas the opposite is expected in the case of frequent and widespread recombination. The understanding of the factors influencing the occurrence and extent of recombination is, therefore, a prerequisite to the development of efficient epidemiological tracking methods and reliable control strategies.

The improvement of population genetics tools during the past thirty years has made it possible to design efficient methods to discriminate and characterize pathogen strains and lineages [12], [19], [20], as well as to estimate the impact of clonality on their evolution [12]–[14], [16].Estimates of recombination rates and identification of recombinant lineages require, however, a large number of neutral markers [19], [20], and accurate phylogenetic reconstructions. Whereas predominant clonality facilitates phylogenetic reconstruction even for recently diverged taxa, the usefulness of classical phylogenetic trees is limited by the occurrence of sporadic recombination. The sporadic exchange of genetic material among long divergent lineages may indeed be more accurately illustrated by a reticulate network than a dichotomic tree. Various methods have been developed to draw networks of haplotypes in order to illustrate uncertainties in mutational pathways separating sequences, or to reconstruct reticulate phylogenies accounting also for reticulate events such as recombination or hybridization [21], [22]. However, to our knowledge, no studies have relied on the application of graph theory-based network analysis (Euler, 1736 in [23]).

Chagas disease is mainly transmitted in America from southern United States to northern Argentina. It affects about 20 million people mostly in Latin America, and it is considered by the World Health Organization as a priority endemia. *T. cruzi*, exhibits predominant clonal evolution [13], [24], with 6 main genetic subdivisions (Discrete Typing Units or DTUs [25] or “near-clades” [14], as previously described [26], [27]. We have coined the term “near-clade” to designate genetic clusters observed in a predominantly clonal population, which cannot be equated to real clades because some occasional recombination interferes at an evolutionary scale [14].

**Figure 1:**
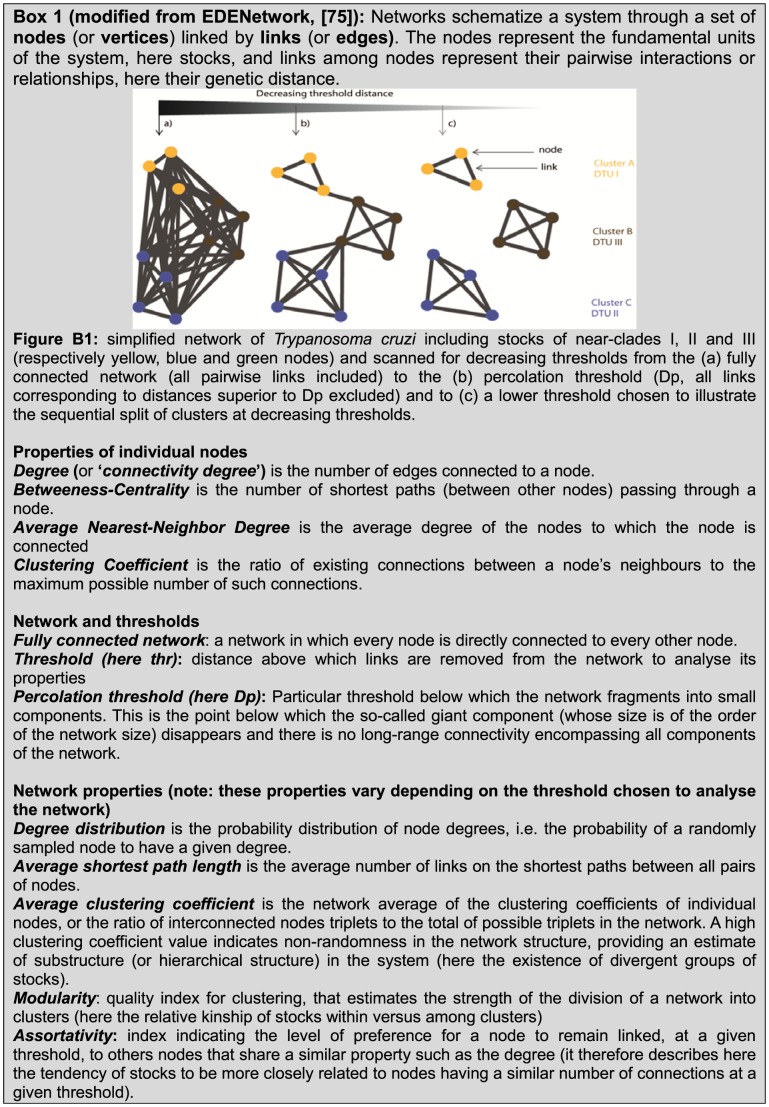
(modified from EDENetwork, [**75**]): Simplified network of *Trypanosoma cruzi* including stocks of near-clades I, II and III (respectively yellow, blue and green nodes) and scanned for decreasing thresholds from the (a) fully connected network (all pairwise links included) to the (b) percolation threshold (Dp, all links corresponding to distances superior to Dp excluded) and to (c) a lower threshold chosen to illustrate the sequential split of clusters at decreasing thresholds, and main properties of nodes and networks used herein.

The 6 *T. cruzi* near-clades have been recently numbered I to VI [28]. They are stable in space and time and represent the relevant lineages for epidemiological tracking and evolutionary studies. Their distribution may vary according to the geographic location, the type of cycle (either domestic or sylvatic) and the host [26], [28], [29]. Despite this predominantly clonal evolution at the level of the whole species, sexual reproduction was recently suggested to occur in southern Ecuador in localized cycles for the near-clade TcI [30], although the evidence presented is questionable [31]. Moreover, experimental recombination has been obtained, with an asymmetric horizontal transfer similar to the ones occurring among bacteria [10]. Lastly, it is widely accepted that some of the *T. cruzi* near-clades have a hybrid origin. Several scenarios of hybridization have been proposed [16], [32]–[35], based on interpretation of phylogenetic trees. Recombination probably plays a crucial rule in the evolution of this pathogen on an evolutionary scale [14], and a clearer picture of the evolution of *T. cruzi* lineages may therefore be obtained using a network, best adapted to represent “genealogies-like” patterns, expected when both parental and daughter lineages are susceptible to be present in the same datasets [32], [33], [34]. Apart from illustrating the reticulate evolution better than a phylogenetic tree, a network can be analysed using network tools specifically developed to better describe the clustering of some groups of lineages (near-clades) and their specific position in the evolution and diversification of near-clades (Figure 1).

*T. cruzi* near-clades exhibit distinct properties, particularly in terms of experimental pathogenicity in mice, transmissibility to the insect vector, and resistance to drugs [36]–[40]. A refined understanding of clonal evolution vs. recombination is therefore important: (a) for the fine characterization and classification of natural isolates, and (b) to improve the understanding of the dynamics and evolution of lineages and genes of interest such as those involved in pathogenicity and drug sensitivity.

In the present study, we have taken into account the impact of occasional recombination on the evolution of *T. cruzi* near-clades by applying a graph theory-based network analysis to the most extensive genetic data available thus far. It consists of 434 isolates sampled across the whole ecogeographical range of *T. cruzi* and characterized by Multilocus Enzyme Electrophoresis (MLEE) at 22 loci [26]. This study has identified 262 MLGs. It has been shown that MLEE variability correlates positively with many other genetic markers [28] and gene expression revealed by proteomic diversity [41], which is strong evidence for linkage disequilibrium (non-random association of genotypes occurring at different loci) and predominant clonal evolution [14]. The results obtained on MLEE data were compared with those obtained from 19 microsatellite loci and 66 MLGs [35]. We have also tested here the recently proposed hypothesis that *T. cruzi* near-clade TcI exhibits a “Russian Doll” pattern; that is to say, it reproduces in miniature the population structure of the whole species, with strong linkage disequilibrium and lesser near-clades [31]. The hypothesis was based on the analysis of previous studies relying on various genetic markers, which had suggested structuration within TcI [42], [43].

## Results

### Clustering and identification of near-clades

The analysis made with the Shared Allele Distance (SAD) standardized between 0 and 1 resulted in a network where nodes represent MLGs and links among them depend on the strength of their pairwise SAD. The networks are scanned for successive thresholds called Dp that can be understood as an estimate of the percentage of shared markers among the stocks and near-clades.

**Figure 2:**
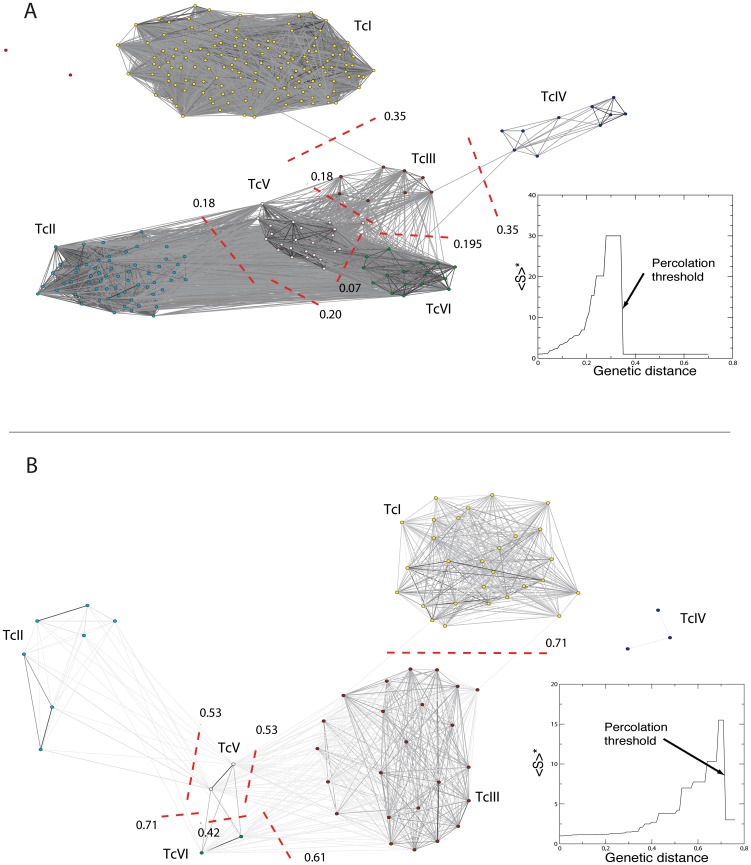
Networks of *Trypanosoma cruzi* based on a) MLEE and b) microsatellites. Networks are represented at the percolation thresholds for each dataset (MLEE: Dp = 0.35; microsatellites: Dp = 0.71). The evolution of the average cluster size (<S>; estimated excluding the largest cluster) is represented on the bottom right of each network. An arrow represents the location on the curve where genetic distance (on the x-axis) corresponds to the percolation threshold (Dp, just before the apparition of the secondary higher cluster, the size <S> of which is projected on the y-axis). Only links with genetic distances (shared allele distance = SAD) smaller than the percolation threshold are represented. A gradient of dark grey to light grey represents decreasing distances among stocks, their relative position have no relationship with their distance but are arranged to minimize the overlap of links and maximize clarity. The threshold at which the six recognized clusters of genotypes (corresponding indeed to the six major near-clades) separate themselves from the most closely related ones are represented by red dashed lines with numbers corresponding to the threshold values. Color code for the near-clades is yellow for TcI, blue for TcII, brown for TcIII, dark blue for TcIV, pink for TcV and green for TcVI.

The network of 262 distinct MLGs from the 434 isolates analysed by MLEE when scanned starting from the percolation threshold Dp = 0.35 (Figure 2a) shows the existence of 6 main clusters corresponding to the main near-clades described in *Trypanosoma cruzi* [28]. The clustering at this threshold (Table 1) is high (<CC> = 0.88) and significantly different from the one expected if links were distributed randomly among nodes (CCo = 0.18, p<10^−3^). This indicates the existence of a hierarchized structure in the dataset.

**Table 1:** Matrix detailing the thresholds below which no links exist between two near-clades, MLEE below diagonal and microsatellites above.

*Near-clades*	*TcI*	*TcII*	*TcIII*	*TcIV*	*TcV*	*TcVI*
***TcI***		0.74	0.72	0.74	0.79	0.83
***TcII***	0.49		0.77	0.81	0.53	0.71
***TcIII***	0.35	0.34		0.75	0.53	0.59
***TcIV***	0.52	0.37	0.35		0.81	0.83
***TcV***	0.41	0.18	0.18	0.36		0.43
***TcVI***	0.47	0.20	0.20	0.35	0.07	

Almost identical results were obtained when scanning the network of 81 stocks analyzed for the 66 MLGs discriminated using 19 microsatellite loci [35], with a similar (<CC> = 0.88) and significant clustering (CCo = 0.40, p<10^−3^) at a much higher percolation point (Dp = 0.71; Figure 2b).

In both cases, the lowest threshold (thr) maintaining ‘among near-clade’ connection (thr = 0.07 for MLEE, Figure S1, 0.42 for microsatellites; Figure 2) corresponds to the persistence of links between TcV and VI when all other near-clades are disconnected. This confirms the genetic proximity between TcV and TcVI. The network can be scanned at decreasing thresholds starting from Dp down to this last point of ‘among near-clade’ disconnection. The clustering is also significant for successive network topologies unravelled through this scan, and clusters disconnect in an order corresponding to their genetic divergence as estimated through the Shared Allele Distance (Figure S1).

Just below the percolation threshold (Dp = 0.35) of the MLEE-based network, the independent splits of the clusters corresponding to TcI and IV (Figure 2a) from the main network mark the disconnection of clusters corresponding to TcII and III, which remain indirectly linked only through stocks of TcV and TcVI. At a lower level, TcVI appears only linked to TcV after thr = 0.195 and the complete isolation of TcV from TcII and TcIII occurs simultaneously below thr = 0.18 (Table 1). The clustering is significant for successive networks scanned in an order following the integration of links corresponding to increasing genetic divergence as estimated with the Shared Allele Distance. This shows how successive clusters connect to each other, until the emergence of the “giant cluster” encompassing all stocks analyzed. The network can be scanned at decreasing thresholds starting from Dp down to this last point of ‘among near-clade’ disconnection. The clustering is also significant for successive network topologies unraveled through this scan, and clusters disconnect in an order corresponding to their genetic divergence as estimated through the Shared Allele Distance (Figure S1).

All stocks were assigned to defined lineages corresponding to clusters disconnecting at increasing thresholds, in a similar ranking for MLEE- and microsatellite-based networks (Table 1). One main difference observed with the microsatellite data is the existence of a dichotomy in the TcII cluster, with one sub-cluster of TcII individualized from the principal cluster (thr = 0.58), while the other one remains more closely related to the remaining TcV and TcIII down to a lower threshold (thr = 0.53). At this point, TcV separates itself simultaneously from both TcII and TcIII, when the only cluster remaining is TcV-TcVI down to thr = 0.425 where the last connected near-clades also split. At this level, TcV also individualizes itself simultaneously from TcII and III (thr = 0.53). TcVI is disconnected from TcII far before (Dp = 71) but separates itself from TcIII at a similar level than TcV (thr = 0.585).

Another exception is a stock recognized as TcVI (Y cl2, Brener) that remains connected to the cluster of TcV according to MLEE data, after having been disconnected from all other stocks of TcVI.

Finally, the Neighbor-Nets phylogenetic networks built also on the basis of ASD distance mainly corroborate the clusters corresponding to the near-clades previously identified, although they also reveal a composite cluster that includes two TcV (Brazil NIH 1954 and 92.80) in the near-clade containing TcVI stocks according to MLEE data. Neighbor-Net results also show numerous “boxes” built by parallel lines identifying the same split or branch and indicating clashing patterns of relationships. Such incompatible splits, numerous in this case both for MLEE and microsatellite data (Figure S2), are expected under the hypothesis of an extensive number of hybridization events (Huson & Bryant, 2006). Besides the illustration of clustering and indices of reticulate events, MLEE NeighborNet showed distances that also support the existence of a cluster TcII,III,V,VI, with TcII appearing more distantly related from the three other near-clades. However, this split appears less clearly on the basis of microsatellite data.

### General properties of the system

The distribution of degree is rather homogeneous for both global networks, together with a lack of correlation between degree (number of links) and clustering, and a positive relationship between the degree of a node (MLG) and the average degree of the connected MLGs. The highest values of betweenness-centrality are consistently observed among genotypes connecting the clusters corresponding to previously recognized near-clades. No relationships were observed between degree and betweenness-centrality.

### Network analysis at the sub-near-clade level

The MLEE network topology for TcI shows a large diversity of interconnected genotypes with an absence of modularity (no significant clustering), illustrated by the lack of emergence of sub-clusters in the network. The low threshold value (Dp = 0.24) at which the percolation is reached illustrates the sharing of more than 75% of alleles among these genotypes.

**Figure 3:**
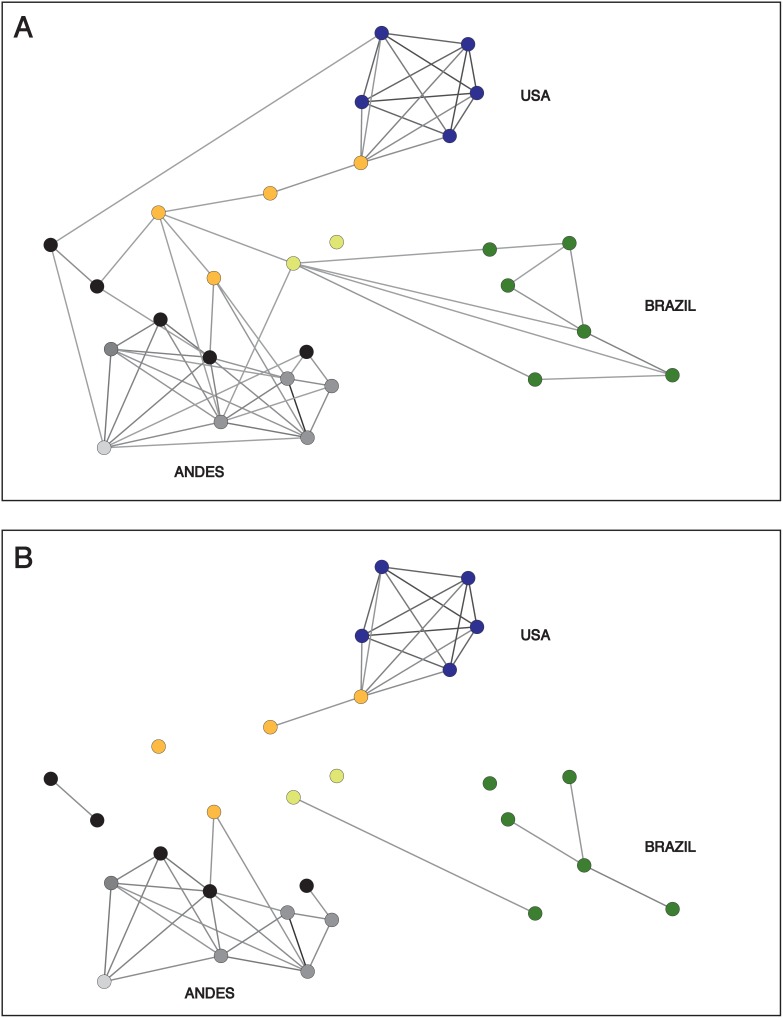
Networks of TcI genotypes characterized with microsatellite data from Lewis et al. (A) Just above percolation distance (Dp = 0.37), and (B) just below, illustrating the disconnection of the 3 clusters of nodes. According to color code, 3 geographical regions are highlighted: USA (Violet), Brazil (Green) and Andes (Peru and Argentina in Dark Grey, Chile in Light Grey, Bolivia in Black). Colombia in Orange and Venezuela in Yellow green show the intermediate position of stocks isolated in those countries, at the interface of the three clusters.

Using the microsatellite data of Lewis et al. [35], three clusters emerged in the network exhibiting significant clustering (<CC> = 0.55, P<10^−3^), essentially corresponding to 3 geographic areas: USA, Brazil and Andes (Argentina, Peru, Chile; Figure 3) linked through Venezuela (M13 and M18), Colombia (458) and Bolivia (Sjmc7 and Sjm32). All three clusters disconnect at the same threshold (Dp = 0.37). Besides the different level of diversity with the two kinds of markers, it should be noticed that only one stock is shared between the 2 datasets (92101601P).

## Discussion

The homogeneous distribution degree, lack of correlation between degree and clustering, and a positive relationship between the degree of a node (genotype) and the average degree of the connected MLGs (a property called *assortativity*) highlight a rather balanced level of connections among stocks in a hierarchically organized system. In fact, the strong modularity of the networks obtained with both MLEE and microsatellite data clearly illustrates the existence of discrete sets of stocks corresponding to the six major near-clades recognized in *Trypanosoma cruzi* [28]. As expected, the network topology also provides a clarified picture of their phylogenetic history, made of both clonal divergence and sporadic hybridization.

Various methods have been developed to draw networks of haplotypes in order to illustrate uncertainties in mutational pathways separating sequences, or to reconstruct reticulate phylogenies accounting also for reticulate events such as recombination or hybridization [21], [22]. It should be underlined that the method proposed here profoundly differs from classical network phylogenetic methods. The rationale behind the network construction and analysis stems from a totally distinct origin, namely graph theory (Euler, 1766 in [23]). Facilitated by computing improvements, this rather old concept has undergone recent development [44], [45]. It has recently been expanded to various fields of biology, including food webs [46], biogeography [47], [48] and population genetics [49]–[52]. Thus far, this graph theory-based network analysis has been used in epidemiology and pathogen evolution only through modelling [41]–[44]. The present analysis is therefore the first application of graph theory-based network analysis to real data concerning a pathogen. Its comparison with an improvement of classical phylogenetic reconstruction to account for reticulate events, the network phylogenies built using the algorithm Neighbor-Net (Huson & Bryant, 2006) shows similar results in terms of clustering (Figure S2), although the relationship among lineages appears clearer on the graph-theory based networks that also allowed the level of significance of clustering to be assessed (Figure 2 and Figure S1). The case of *Trypanosoma cruzi* is exemplary for the application of such kind of analysis to the evolution of clonal lineages. The identification by pioneering MLEE studies of 3 “zymodemes” (sets of isolates sharing the same MLEE profile) [53] has been further completed, and the evolutionary nature of the zymodemes, which correspond to clonal lineages undergoing occasional bouts of recombination, have been elucidated by population genetics means [13], [14], [24]. A reunified nomenclature has been recently proposed [28] confirming the existence of 6 major subdivisions [26], [27], [31] (TcI to VI) or near-clades [14]. The results obtained here replaced in the perspective of these recent developments provides additional support to these proposed subdivisions through the analysis of graph theory-based network clustering and modularity, while they allow to propose new scenarios that emerge from the network topology. These results illustrate the potential of graph theory-based network analysis to characterize pathogen lineages, discriminate major groups with potentially distinct clinical and epidemiologic properties, and retrace the history of their emergence including complex and ancient events of hybridization among pathogen lineages.

### Clustering and identification of near-clades

The discrimination of near-clade appears clearly at the percolation threshold on network (Figure 2), with the single exception of TcVI Brener that remains clustered with TcV instead of TcVI at the lowest threshold according to MLEE data. This is possibly due to a high variance level for low distances, and the assignment appears correct with the microsatellite dataset, for which less stocks are available. The distinction between TcV and TcVI is unclear with NeighborNet on the MLEE dataset (Figure S2) as well as with Girvan Newman community analysis (Figure S3). The relative divergence among near-clades however appears clearly when scanning the networks at increasing thresholds, which makes it possible with this method to distinguish between clustering and divergence patterns that result from the most recent events of diversification and emergence on one hand, and the most ancient events on the other hand. The quantitative difference of Shared Allele Distances observed between MLEE and microsatellites (Table 1) is a likely consequence of the higher mutation rate of the later [54], possibly combined with lower stabilizing selective influence, resulting in stronger signatures of divergence among stocks and near-clades with microsatellites at micro-evolutionary levels. Qualitatively however, both networks retrace similar histories of genotype clustering, and divergence among near-clades (Figure 2&3), an information that appears less clear on the unique snapshots offered by Neighbor-Net (Figure S2) or community analysis (Figure S3).

The delimitation of the six recognized near-clades is confirmed by the formation of clusters connecting their stocks as only the lowest distances are introduced into the networks (Figure 2&3, darkest links). This shows a relatively long-term independent divergence of stocks belonging to distinct near-clades. In fact, the first links among near-clades appear between TcV and VI at thr = 0.09 for MLEE and thr = 0.43 for microsatellites (Figure 2&3; Table 1), once within-near-clade genotypes are fully linked (Figure 2&3).

This first connection is consistent with our present knowledge, since TcV and TcVI are the most recently emerged near-clades. A recent phylogenetic analysis that used a molecular clock has estimated their time of emergence at 60,000 and 30,000 years ago [35], respectively, following a similar event of hybridization between TcII and TcIII [32], [34]. By increasing the threshold and adding links that correspond to increasing Shared Allele Distances, the evolving network topology corroborates the hypothesis of either the same or two similar events involving the same parental lineages. Connection between the two near-clades TcV and TcVI occurs first, then TcIII and TcII are added to this cluster–first through connection with TcV- at similar thresholds (Dp = 0.18 and Dp = 0.53 for MLEE and microsatellites, respectively). Finally, at the percolation threshold (Dp = 0.35 and Dp = 0.71; Table 1) or slightly higher (Dp = 0.36), when the highest cluster encompassing all near-clades is connected, the super cluster TcII+III+V+VI connects itself to TcI and TcIV through TcIII (Figure 2&3). This late connection of the clusters corresponding to TcI and TcIV to the main network illustrates their higher divergence from the other near-clades.

Although much progress has been made during the last decade in the study of *T. cruzi* molecular evolution, with increasingly variable markers, no unequivocal, reproducible and standardized method was available to “*simultaneously distinguish the known genetic lineages, describe inter-DTU relationships, and define high resolution intra-DTU diversity for population genetics studies*” [17]. The MLST approach proposed by Yeo et al. [17] aimed at reaching this goal ([17], see also [55]). Based on both MLEE and microsatellite typing, the SAD networks presented here fully support the individualization of the previously recognized near-clades. This method thus constitutes a reliable tool, provided that a sufficient number of loci are used, to assign a new genotype to a given near-clade, a first step towards any inferences about its clinical and epidemiological properties. Such assignment can apparently be made without ambiguity, at least with the stocks analyzed here, with the exception of TcVI-Brener when MLEE data are concerned. Within-near-clade genotypes are connected by strong links at low threshold distance (Figure 2&3), compared to weaker links appearing at higher distances that connect distinct near-clades.

The clear delimitation of near-clades revealed by strong clustering and modularity of the network, confirms the scarce nature of genetic exchange among near-clades, and is in agreement with the hypothesis that they are the result of predominant, long-term clonal evolution [13], [14], [28], [31].

The strong correlation between different markers (MLEE and microsatellites), and the fact that network analysis uncovers the same clusters as classical phylogenetic analysis confirms the existence in *T. cruzi* of near-clades that are stable in space and time. We have postulated [14] that such a robust clustering, corroborated by different approaches and accumulating evidence (congruence principle, [56]) characterizes predominant clonal evolution, together with a strong linkage disequilibrium.

We discuss below the potential of network analysis to describe inter-near-clade relationships, reveal intra-near-clade diversity and infer their history of divergence and diversification.

### Near-clade history of hybridization

Hybridization events are hypothesized to have occurred during the evolution of *T. cruzi* near-clades. The first scenario proposed by Westenberger proposed a sequence of two hybridization events. The most ancient event is supposed to have occurred between TcI and TcII to give rise to TcIV and TcIII. The most recent hybridization, between TcII and TcIII, would have resulted in the emergence of TcV and TcVI [32], [35]. Although most subsequent studies based on MLST supported the second hybridization event, the hypothesis of the ancestral hybridization event having resulted in TcIII and IV has been seriously questioned. One study based on both mitochondrial and nuclear genes and suggesting a third ancestral lineage at the origin of TcIII and a more complex and still unravelled origin for TcIV [57]. Another study, based on the analysis of 32 nuclear and mitochondrial unlinked loci, also failed to support the hybrid origin of TcIII and TcIV as well as the dichotomy corresponding to the originally defined two major lineages TcI and TcII [58]. The topology of the networks built in the present study clearly supports the hypothesis of the more recent hybridization event proposed by Westenberger et al. [32] and supported by more recent studies, whereas different interpretations of networks can be suggested in relation to the hypothesis of an ancient hybridization event at the origin of TcIII and TcIV.

While the nearly identical distance between TcIII and TcIV on one hand, and TcI and TcII on the other hand, is expected in the case of an hybridization event (Figure 2, Table 1, Table S1 & Table S2), it may also support the hypothesis of a nearly simultaneous divergence of their ancestors. Now, when screening more carefully the relationship among these near-clades, TcIV appears almost equally distant to the two large subdivisions TcI and TcII-III-V-VI emerging at percolation when analyzed with microsatellites data, but clusters preferentially with TcII-III-V-VI when MLEE data are concerned (Dp = 0.35 versus Dp = 0.52 for a connection with TcI; Table 1). This supports similar observations based on MLST [17]. The different connection patterns of TcIV with either microsatellites or MLEE may be due to the variance in distance estimates explainable by the fact that a smaller set of stocks have been used with microsatellites [35] than with MLEE [26]. Differences based on the *Gpi* nuclear gene [18], as well as on cytometric [33] or genetic analysis with distinct microsatellite loci [33], [59], were shown to be important between TcI and TcIV. Nevertheless, some mitochondrial sequences belonging to strains isolated from opossums in the USA and unambiguously identified as TcI with nuclear markers, were shown to be nearly identical to TcIV. Such great incongruence is likely explainable by a mitochondrial introgression between distantly related near-clades [35]. It is nevertheless thought that these events have been too scarce to have a strong influence on the evolution of *T. cruzi* [60]. In line with these observations, the present network analysis supports the hypothesis of a closer proximity between TcIV and TcI with microsatellites than with MLEE. This could be due possibly to ancient asymmetrical exchanges. Finally, the network topology mostly distinguishes four groups, including TcI, TcII, TcIII and TcIV, linked together through TcIII (for Tc I and TcIV) and TcV (for TcII and TcIII). This topology is therefore also compatible with the hypothesis of the evolution of three major lineages (TcI, TcII and the ancestor of TcIII-TcIV) having later led to the hybridization of two of them (TcII and TcIII), a hypothesis also favoured by more recent sequence analysis [61].

In both networks in the case of TcV and in the MLEE network in the case of TcVI, links to the putative parental near-clades TcII and TcIII appear simultaneously at similar thresholds (Table 1), and the cluster TcV-TcVI thus builds a bridge between the two divergent near-clades TcII and TcIII. Such a striking equidistance illustrates the similar amount of genetic material shared with both parental near-clades. Rather than a chaotic lateral transfer sometimes hypothesized and often observed in bacteria [62], [63], this topology is in agreement with the hypothesis of an origin of hybridization through polyploidization followed by progressive but rather homogeneous diploidization or aneuploidization [10], [16], [32], [34], [57], [64], as well as with recent estimates of private and shared alleles among these near-clades with the microsatellite dataset used here [35]. While it is still unknown whether TcV and TcVI have derived from the same hybridization event or from two distinct hybridization events involving the same parental near-clades as suggested on the basis of the same microsatellite dataset [35], joint network topologies together with the amount of shared polymorphism among TcV and TcVI fits better the first hypothesis. As a matter of fact, in the case of the second hypothesis, one would expect less proximity of the two “daughter” near-clades and a higher variance in their connections with parental near-clades, since not necessarily the same half of parental genome would be shared (Table 1, Table S1). Besides, TcVI connects to putative parents first through TcV, suggesting either a possible founder effect at the origin of this lineage having led to a closer relationship with TcV, or a backcross origin involving TcV and TcIII early in the history of these hybrid lineages. Both hypotheses may explain the inconsistency in the position of two TcVI strains, including TcVI CL-Brener above mentioned, which are linked either to TcVI or TcV according to the dataset considered.

Finally, the numerous incompatible splits observed with NeighborNet both for MLEE and microsatellite data (Figure S2), are expected under the hypothesis of an extensive number of hybridization events [21]. In the particular case of partially clonal pathogens, we suggest that they may however be the result of a limited number of hybridization events followed by the independent evolution of lineages within each near-clade, particularly in cases where the species considered evolve through predominant clonal evolution and therefore preserve balanced proximity with both parental genome.

### Scarce genetic exchange, high imprint of clonality

**Figure 4:**
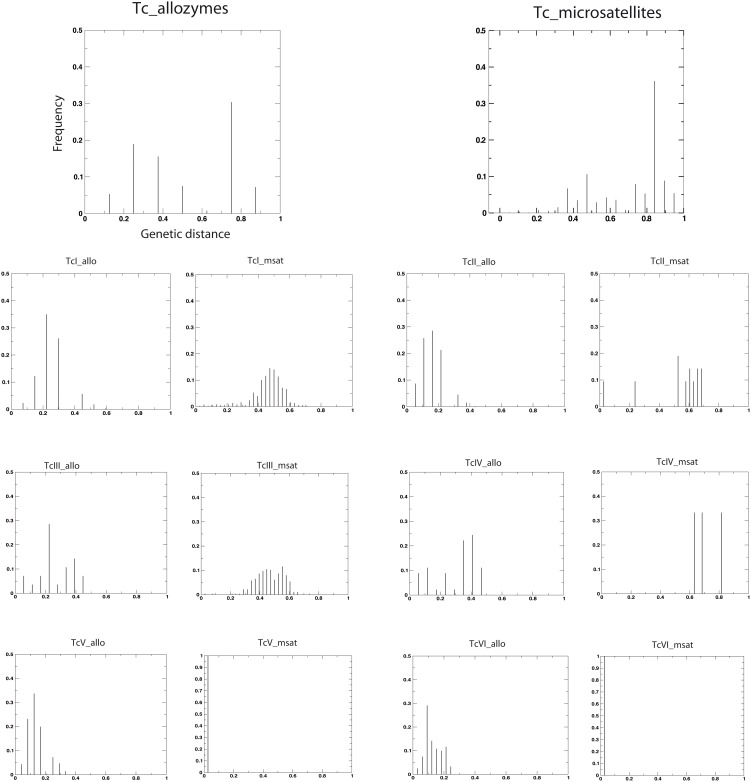
Genetic Distances Spectrum. The frequency distribution of genetic distances among all stocks (upper line) and among stocks within-near-clade (below) using Shared Alleles Distances with microsatellite (right) and MLEE (left) data.

The importance of clonality in the evolution of *T. cruzi* lineages is confirmed by several properties of the networks. The balance of connectivity degree distribution (and assortativity) within each of the six robust clusters identified here suggests waves of nearly synchronized events of within-near-clade divergence, preceding long term diversification through predominant clonal evolution. This is particularly well illustrated by the bimodal frequency distribution of MLEE distances within TcII, TcIII, TcIV and TcV (Figure 4), resulting in significant clustering in the corresponding networks and suggesting: i) the occurrence of an ancient, synchronized event of diversification among stocks separated by very similar genetic distance (second peak with a wave of similar distances); and ii) the occurrence of diversification through the recent accumulation of somatic mutations (first peak at lower distance).

Intra-near-clade structuring, when analyzed with a number of stocks which is large enough to reliably reveal it, is especially clear in the analysis of Genetic distance spectrum (GDS) or network representing the frequency distribution of distances among stocks, for microsatellite data (Figure 3 & 4). This is likely due to a higher power of resolution of microsatellites due to their higher evolution rate. Both GDS analysis (Figure 4) and network of TcI with microsatellites (Figure 3) support scarce genetic exchange among differentiated clusters within near-clades (“Russian doll pattern”; [31]). Clusters emerging in the network of TcI show a segregation of stocks in line with their geographical origin (Figure 3). This suggests that, in the case of this stock sampling, geographic isolation may in part explain the long-term divergence among differentiated clusters of the same near-clade, and dispersion may be limited enough at the scale of the American continent to induce vicariance and clonal divergence. However, the Russian doll pattern seen within TcI through various other studies show that the lesser near-clades within this near-clade do not follow a model of isolation by distance. Some of them are ubiquitous and widespread, and they may occur sympatrically [31].

Studies of hybridizing lineages of partially clonal algae have already shown the usefulness of network analysis to disentangle the influence of ancestral polymorphism and present introgression through hybridization [50]. The results exposed here, although they deal with a distinct system where suspected hybridization events are very ancient, illustrate in a similar manner the power of network analysis to assign lineages to a given pathogen cluster or “near clade” [14] with well characterized distribution and properties, and to elucidate past events of introgression. This study shows how data from very classical markers can be updated and more precisely analyzed by appropriate methods. This study opens promising prospects for the use of network analysis to unravel biogeographical and evolutionary patterns and relationships among pathogen lineages. As a matter of fact, many pathogens, if not most, including viruses, bacteria, fungi and parasitic protozoa, show common points with *T. cruzi* evolution, namely preponderant clonal evolution with occasional genetic exchange, and near-clading [14], [31]. The added value of the method is underlined by the fact that we have used ancient data analyzed with other, more classical methods (usual phylogenies). The present results clearly show that with the same experimental data and the same sets of stocks, graph theory-based network analysis evidences far more refined results.

The method proposed here will help inferring pathogen clinical and epidemiological properties through a phylogenetic character mapping approach [41].

## Materials and Methods

### Origin of the stocks & Experimental conditions

The stocks characterized by MLEE have been presented in reference [26] and include 434 isolates delivering 262 distinct MLEE discriminated using 22 allozyme loci. They have been isolated over vast geographical ranges and spans of time, from a diversified panel of hosts, including various mammal and triatomine bug species. As underlined recently [14], such a sampling at highest time and space scales is the best adapted to analyze the overall population structure of a given species.

The microsatellite dataset from Lewis et al. [35] was additionally analysed for comparative and corroboration purposes, since this is the most recent and complete one presently available. We chose to analyse the dataset with 19 loci published in Lewis et al.'s [35]study, as it allowed reconstructing a network with a larger number of stocks (81), resulting in 66 distinct MLG.

In order to explore the feasibility of biogeographical analysis at the infra-near-clade level, we also explored the networks of MLEE and microsatellites for the only near-clade TcI using the same data, including therefore 144 distinct MLGs for MLEE and 28 distinct MLG for microsatellite data.

### Network construction

The networks characterized for MLEE (262 MLGs) and microsatellites (66 MLGs) were built separately and compared after independent, yet identical, analysis based on graph theory [65]. A network is represented by a graph consisting of two sets: nodes and links that illustrate the pairwise relationships among nodes. In this study, nodes correspond to each of the *T. cruzi* MLGs in the datasets for i) MLEE; and ii) microsatellites, and links represent the genetic distance between them.

The Shared Allele distance (SAD) was used as genetic distance and calculated based on MLEE data. This genetic distance measures the proportion of shared alleles [66]. For stock pairwise comparisons, the proportion of shared alleles is estimated by:
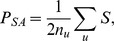
where the number of shared alleles S is summed up over all loci u, and n_u_ is the number of loci

Distance between nodes,
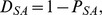



This measure can be used to look at population substructure. Bowcock et al. [67] have constructed dendrograms based on this distance calculated from human microsatellite data. This distance measure has also proved to be very successful at placing unknown individuals into the correct subpopulation [68] or at characterizing hybridization phenomena in sexual, diploid algae [50]. The computation of the adjacency matrix (AM) of genetic distances among genotypes with values ranging between 0 (identical genotypes) and 1 (no alleles in common) is the first step for network construction.

A fully connected network linking all genotypes is then built, and a relevant criterion has to be chosen in order to take into account the existence of connectivity between each pair of nodes. This step is crucial for the resolution of the system dynamics and its modularity. The strategy used in this study is based on the percolation theory [69] that allows the splitting of the fully-connected network into discrete clusters of nodes [48], [50], [52] on the basis of inner properties of the network. Links are sequentially removed starting from those corresponding to the largest distances, with the aim to identify the threshold of genetic distance at which the network studied is a minimal higher cluster composed of sub-clusters linked through primary connections. Below this distance, the network collapses into disconnected sub-clusters, and the pattern of global connectivity is lost.

This critical threshold distance is also named percolation distance (Dp). For a finite system, this point is derived by calculating the average cluster size of all clusters excluding the largest one,
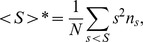
which depends on the last threshold distance value beyond which links were removed. N is the total number of nodes not included in the largest cluster, Smax is the size of the largest cluster and n_s_ is the number of clusters containing s nodes. The Dp is then heuristically identified in the transitional region characterized by a strong decrease in <*S*>* (the average size of these “secondary” clusters excluding the largest one). The network topology and its characteristics are analyzed at this Dp percolation distance, meaning that the links retained in the network analyzed are the ones corresponding to genetic distances lower than, or equal to, Dp, while all links beyond this value are discarded. Additionally, the network is also scanned and analyzed at different sequentially decreasing distance thresholds around this percolation point, in order to assess the consistency of its topology and the inferred properties and interpretation.

For comparative proposes, NeighborNet planar graphs of SAD distances between stocks were constructed with SplitsTree 4.1 [21].

### Network analysis

Networks are used to help understanding the structure and the dynamics of a system of interactions (Figure 1). There are a series of measures [70] that allow characterizing the network topology and interpret it in terms of information flow (here genetic similarity reflecting past common history/ancestor) through the agents (here the genotypes). For example, the *connectivity degree*, *k_i_* of a given node *i*, is the number of other nodes linked to it (i.e., the number of neighbor nodes). The number of links existing among the neighbors of node *i*, is called *E_i_*. This quantity takes values between 0 and *E_i_* ^(max)^ = *k_i_*(*k_i_*−1)/2, which is the case in a fully connected neighborhood. This value is used to calculate the *clustering coefficient C_i_* of node *i*, defined as:
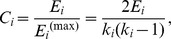



*C_i_* quantifies how close the node *i* and its neighbors are to being a clique. The *clustering coefficient* [44] of the whole network <*CC*> is defined as the average of all the individual clustering coefficients in the system. *C_i_* values vary between 0 and 1. The *clustering coefficient* informs about the hierarchical organization of nodes into distinct clusters (here of stocks into near-clades). In order to test the existence of such sub-structuring, i.e. the significance of <*CC*>, its significance is assessed by comparing the value in the real network to the average value <*CC_o_*> of 1,000 randomized networks obtained by randomly rewiring the number of links present at the chosen threshold among nodes.

The *betweenness centrality* [71] of node *i*, *bc*(*i*), counts the fraction of shortest paths between pairs of nodes that pass through node *i*. Let σ_st_ denote the number of shortest paths connecting nodes *s* and *t*, and σ_st_(*i*) the number of those passing through the node *i*; then,
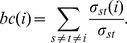



The *betweenness centrality* determines the relative importance of a node within the network as an intermediary in the flow of information. In the case of *T. cruzi*, high betweeness centrality is expected to be observed if hybridization has occurred, in those stocks or near-clades that would have an intermediate position because they would be either “parental” or “offspring” lineages and therefore exhibit a central position among other stocks or near-clades.

The *path length* between any two nodes is defined as the minimal number of steps (links) separating them. The diameter *L* of the network is the maximal path length present in the network. Finally, the density of links *r* is the ratio between the actual number of links present in the network and the number of links in a fully connected network [i.e., *N*(*N*−1)/2].

Finally, the *modularity* properties of the network are also investigated through the Newman and Girvan algorithm [72]. Modularity is a quality index for clustering, that estimates the strength of the division of the network into modules (i.e. clusters, or communities). Strong and significant structure with high values of modularity, emerges when the nodes within clusters share dense internal connections while only sparse links are observed between different clusters. The number of communities is assessed and tested against the same null model as other parameters (see here-below). Networks are visualized and analysed using the Pajek software [73].

In order to test the significance of the topology of the network, 1,000 networks were generated distributing randomly the same number of links between the nodes while keeping the degree distribution observed in the original network [74]. The random distribution of each parameter describing the network was then built on the basis of those 1,000 simulated random networks, in order to test for the significance of the original parameters by assessing their departure from this random distribution. Random simulations were compiled by C++ scripts.

The method presented here is now available to a broad spectrum of users through a user friendly software which was completed recently to allow building networks with several distances (including the distance chosen here), which choice will depend on the dataset analyzed and the questions to be addressed. Using this software it will be possible to both draw networks and analyze their properties as performed here [75].

## Supporting Information

Figure S1 **Illustration of the MLEE network scanning at decreasing thresholds, from the distance threshold 0.70 to 0.06.** On the central curve detailing the evolution of the average cluster size (<S>; estimated excluding the largest cluster, is projected on the y-axis) as a function of the genetic distance (on the x-axis), arrows indicate the threshold chosen for each of the four network represented. Only links with genetic distances (shared allele distance = SAD) smaller than the chosen threshold (indicated below each network) are represented. Color code for the near-clades is yellow for TcI, blue for TcII, brown for TcIII, dark blue for TcIV, pink for TcV and green for TcVI.
(TIF)

Figure S2 **NeighborNet illustrating the reticulated relationship among stocks of Trypanosoma cruzi.** The upper panel shows results for MLEE and the lower panel shows results for microsatellites. Color code for the near-clades is yellow for TcI, blue for TcII, brown for TcIII, dark blue for TcIV, pink for TcV and green for TcVI.
(TIF)

Figure S3 **Communities detected using Girvan-Newman algorithm on allozymes.** The identification of several clusters or modules (M) is illustrated A) at percolation distance (0.63) and B) at the lowest threshold before complete disconnection. (0.07). Color code for the near-clades is yellow for TcI, blue for TcII, brown for TcIII, dark blue for TcIV, pink for TcV and green for TcVI.
(TIF)

Table S1 **Stocks bearing the first link connecting clusters forming near-clades with allozymes.**
(DOCX)

Table S2 **Average genetic distance and confidence interval (CI95) of intra and inter T. cruzi lineages inside the matrix distance.** A: Genetic distance based on Allozymes and B: Generic distance based on Microsatellites.
(DOCX)
