# Supplementary figures and images for: Discriminating Micropathogen Lineages and Their Reticulate Evolution through Graph Theory-Based Network Analysis: The Case of Trypanosoma cruzi, the Agent of Chagas Disease
Source: PLoS One. 2014 Aug 22;9(8):e103213. doi: 10.1371/journal.pone.0103213 (PMC4141739; doi:10.1371/journal.pone.0103213)

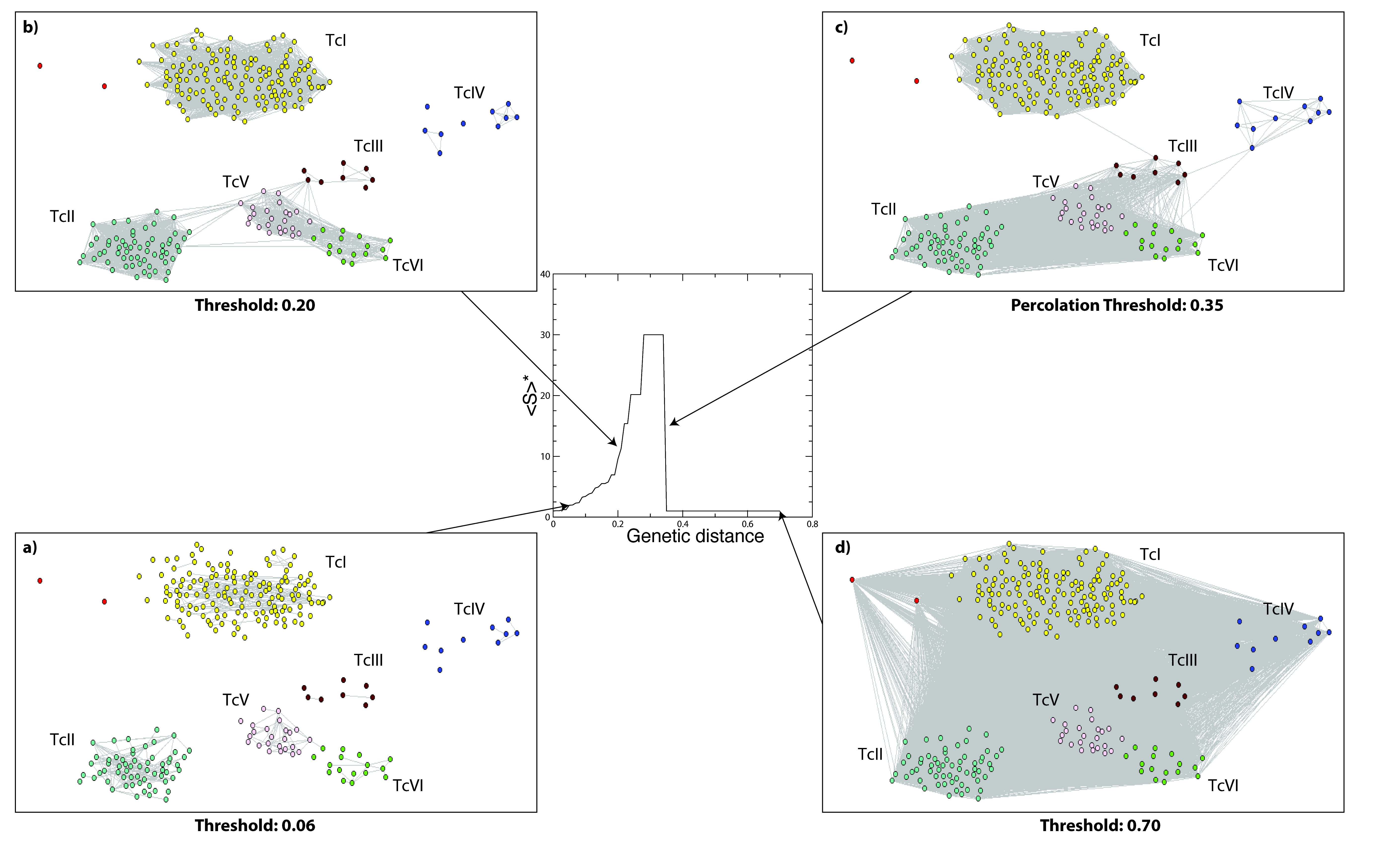

Supplement: Figure S1 — Illustration of the MLEE network scanning at decreasing thresholds, from the distance threshold 0.70 to 0.06. On the central curve detailing the evolution of the average cluster size (; estimated excluding the largest cluster, is projected on the y-axis) as a function of the genetic distance (on the x-axis), arrows indicate the threshold chosen for each of the four network represented. Only links with genetic distances (shared allele distance = SAD) smaller than the chosen threshold (indicated below each network) are represented. Color code for the near-clades is yellow for TcI, blue for TcII, brown for TcIII, dark blue for TcIV, pink for TcV and green for TcVI. (TIF) [file pone.0103213.s001.tif]

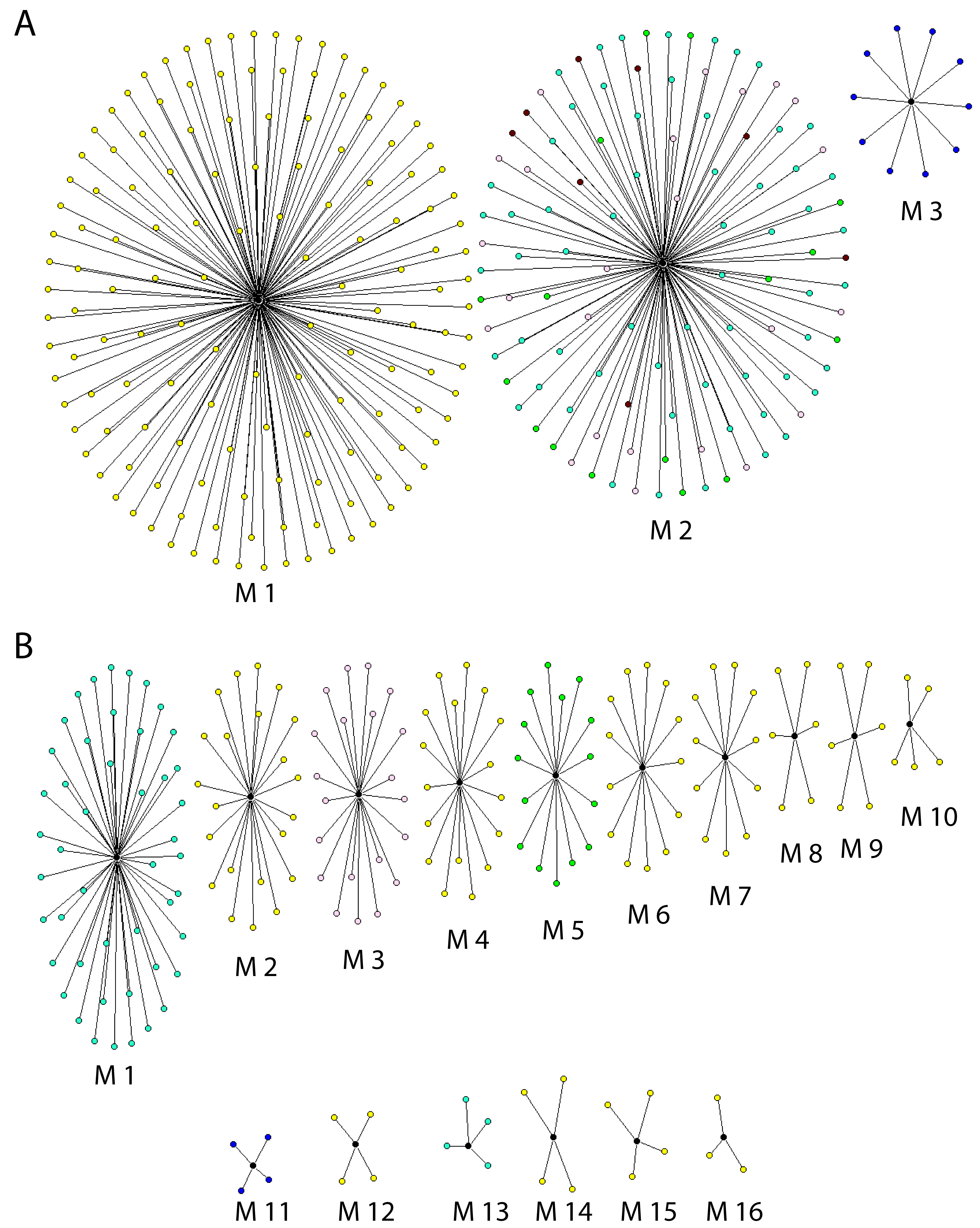

Supplement: Figure S3 — Communities detected using Girvan-Newman algorithm on allozymes. The identification of several clusters or modules (M) is illustrated A) at percolation distance (0.63) and B) at the lowest threshold before complete disconnection. (0.07). Color code for the near-clades is yellow for TcI, blue for TcII, brown for TcIII, dark blue for TcIV, pink for TcV and green for TcVI. (TIF) [file pone.0103213.s003.tif]
