# Supplementary material for: Discriminating Micropathogen Lineages and Their Reticulate Evolution through Graph Theory-Based Network Analysis: The Case of Trypanosoma cruzi, the Agent of Chagas Disease
Source: PLoS One. 2014 Aug 22;9(8):e103213. doi: 10.1371/journal.pone.0103213 (PMC4141739; doi:10.1371/journal.pone.0103213)
Supplement: Table S1 — Stocks bearing the first link connecting clusters forming near-clades with allozymes. (DOCX) [file pone.0103213.s004.docx]

Table S1: Stocks bearing the first link connecting clusters forming near-clades with allozymes.

| **DTUs** | **TcI** | | **TcII** | **TcIII** | **TcIV** | **TcV** | **TcVI** |
| --- | --- | --- | --- | --- | --- | --- | --- |
| **TcI** |  | 0.74 | | 0.72 | 0.74 | 0.79 | 0.83 |
| **TcII** | TLC* |  | | 0.77 | 0.81 | 0.53 | 0.71 |
| **TcIII** | CM25cl2-Xe5012 | CM25cl2-Chaco23Col4 | |  | 0.75 | 0.53 | 0.59 |
| **TcIV** | (10R26; CanIIIcl)-M7 | 10R26-CBBcl2 | | 10R26-Armac13cl1 |  | 0.81 | 0.83 |
| **TcV** | (Chaco2cl3, PAH179cl5)-Xe5012 | Pah179cl5-(IVVcl4,Tu18cl2,CBBcl2) | | (Chaco2cl3, PAH179cl5)-Arma13cl1 | (Chaco2cl3, PAH179cl5)-10R26 |  | 0.43 |
| **TcVI** | (P251cl7, Chaco9col15)-M18cl4 | (P251cl7, Chaco9col15)-(T665cl1,Chaco23col4) | | (P251cl7, Chaco9col15)-Arma9 | (P251cl7, Chaco9col15))-10R26 | (P251cl7, Chaco9col15)-Chaco2cl3** |  |
| * Two large clusters connected, splitting simultaneously  **CL Brener F11F5 connects first to TcV yet before the cluster TcVI is formed | | | | |  |  |  |
