# Supplementary material for: Discriminating Micropathogen Lineages and Their Reticulate Evolution through Graph Theory-Based Network Analysis: The Case of Trypanosoma cruzi, the Agent of Chagas Disease
Source: PLoS One. 2014 Aug 22;9(8):e103213. doi: 10.1371/journal.pone.0103213 (PMC4141739; doi:10.1371/journal.pone.0103213)
Supplement: Table S2 — Average genetic distance and confidence interval (CI95) of intra and inter T. cruzi lineages inside the matrix distance. A: Genetic distance based on Allozymes and B: Generic distance based on Microsatellites. (DOCX) [file pone.0103213.s005.docx]

| B | **TcI** | **TcII** | **TcIII** | **TcIV** | **TcV** | **TcVI** |
| --- | --- | --- | --- | --- | --- | --- |
| **TcI** | *0.459±0.011* | 0.900±0.005 | 0.854±0.004 | 0.854±0.004 | 0.879±0.009 | 0.899±0.009 |
| **TcII** |  | *0.519±0.088* | 0.887±0.006 | 0.877±0.018 | 0.618±0.040 | 0.744±0.013 |
| **TcIII** |  |  | *0.467±0.012* | 0.901±0.011 | 0.639±0.015 | 0.694±0.014 |
| **TcIV** |  |  |  | *0.711±0.107* | 0.895±0.042 | 0.886±0.029 |
| **TcV** |  |  |  |  | *0.026** | 0.434±0.015 |
| **TcVI** |  |  |  |  |  | *0.026** |

**TABLE S2:** Average genetic distance and confidence interval (CI95) of intra and inter *T. cruzi* lineages inside the matrix distance. A: Genetic distance based on Allozymes and B: Generic distance based on Microsatellites.

*:No CI95, estimates based on a single value

| A | **TcI** | **TcII** | **TcIII** | **TcIV** | **TcV** | **TcVI** |
| --- | --- | --- | --- | --- | --- | --- |
| **TcI** | *0.223±0.011* | 0.707±0.001 | 0.588±0.004 | 0.588±0.004 | 0.611±0.002 | 0.662±0.002 |
| **TcII** |  | *0.151±0.004* | 0.470±0.007 | 0.502±0.013 | 0.360±0.003 | 0.366±0.004 |
| **TcIII** |  |  | *0.239±0.012* | 0.502±0.011 | 0.309±0.010 | 0.372±0.012 |
| **TcIV** |  |  |  | *0.276±0.038* | 0.517±0.006 | 0.519±0.010 |
| **TcV** |  |  |  |  | *0.119±0.008* | 0.243±0.006 |
| **TcVI** |  |  |  |  |  | *0.118±0.010* |
